# Adrenal schwannoma can be FDG-Avid on PET/CT: case report and review of historic institutional pathology

**DOI:** 10.1186/s13000-023-01399-5

**Published:** 2023-10-13

**Authors:** Oliver J. Fackelmayer, Eduardo D. Rodriguez, Anthony E. Sisk, Masha J. Livhits

**Affiliations:** 1 Section of Endocrine Surgery, University of California, Los Angeles, Los Angeles, CA 90095 USA; 2 Divsion of General, Endocrine and Metabolic Surgery, University of Kentucky, Lexington, KY 40508 USA; 3 Department of Pathology, University of California, Los Angeles, Los Angeles, CA 90095 USA

**Keywords:** Adrenal gland, Adrenal schwannoma, Adrenalectomy, Adrenal nodule, Adrenal Tumor

## Abstract

Schwannomas are benign, generally indolent tumors of neural crest origin and comprise the most common histologic tumor of peripheral nerves. Schwannomas are a rare histology for retroperitoneal tumors and very rare histologic findings for tumors of the adrenal gland with fewer than 50 cases in the reported literature. Here we present a case report of a non-hormonally functional but metabolically active adrenal tumor with indeterminate imaging characteristics with final pathology showing a 6.1 cm adrenal schwannoma as well as historical institutional pathology review revealing two additional cases.

## Case report

A fifty-year-old male patient underwent routine laboratory analysis during an annual primary care visit which was significant for a mild transaminitis. He subsequently underwent an abdominal ultrasound noting hepatic steatosis and an incidental 6 cm left upper quadrant mass. His past medical history is significant for hypertension controlled with combination lisinopril/hydrochlorothiazide, obesity with a BMI of 39, and current smoker. He had no prior surgical history. His family history was significant only for his mother with uterine cancer.

**Fig. 1:**
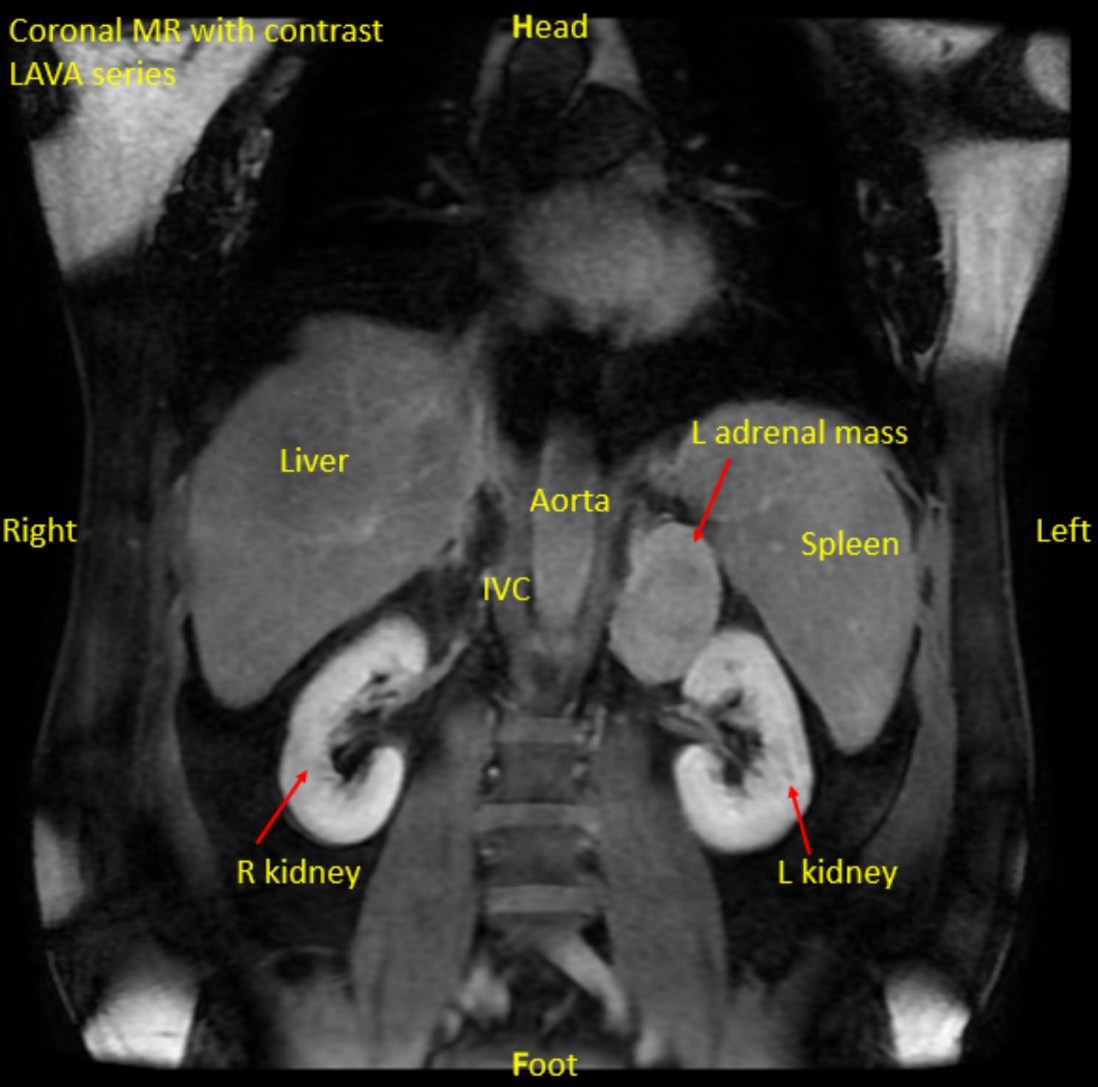
MRI abdomen with contrast, coronal, labeled

He was subsequently evaluated with an MRI abdomen revealing a 6.8 cm well-circumscribed but heterogeneously enhancing left adrenal mass concerning for pheochromocytoma or adrenocortical carcinoma (Fig. 1).

**Fig. 2:**
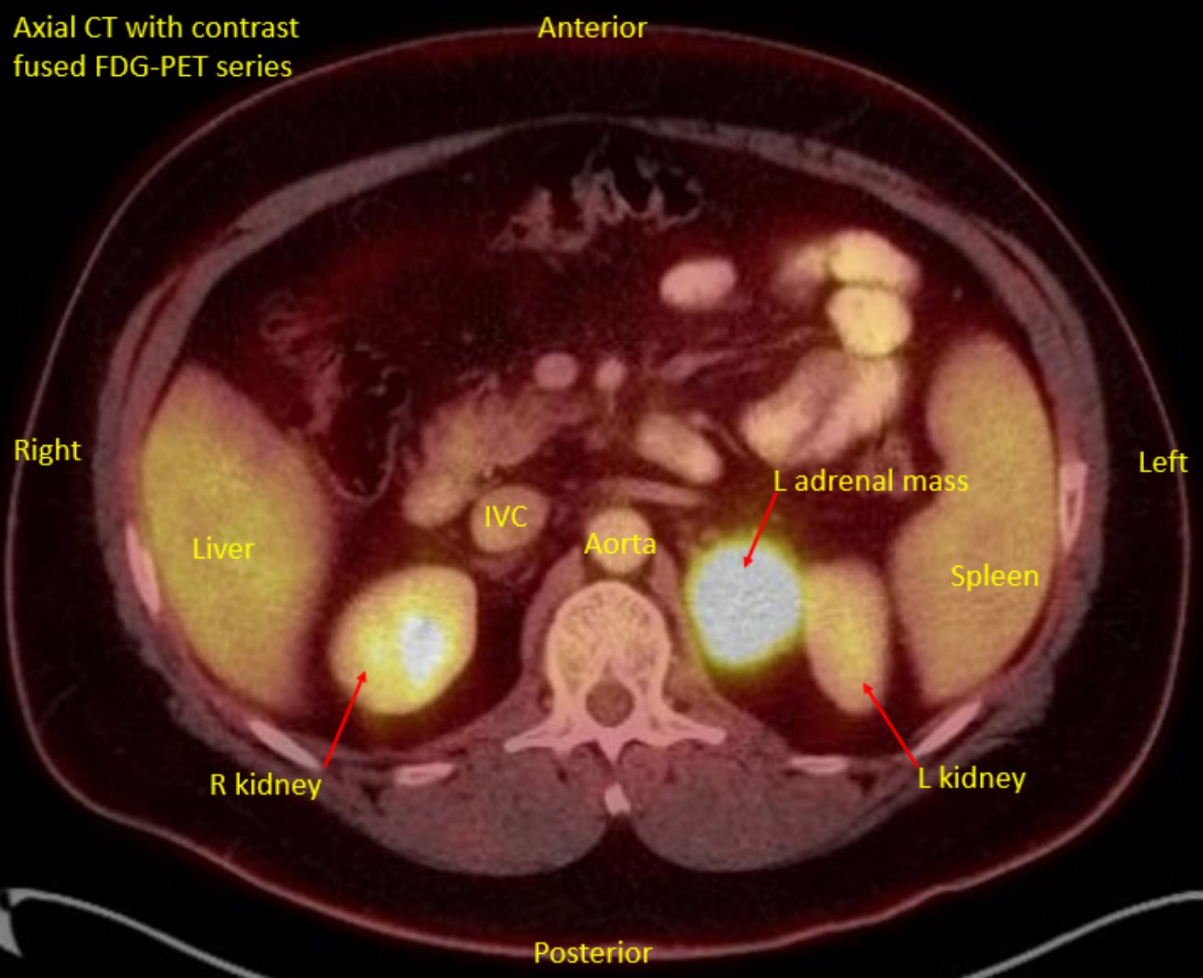
FDG-PET/CT, axial CT fused, labeled

Given the size and indeterminate imaging characteristics of the adrenal mass, he underwent an FDG-PET/CT which showed the mass to be intensely FDG-avid without other areas of non-physiologic uptake (Fig. 2).

His functional workup was significant for normal levels of cortisol, potassium, aldosterone, plasma renin activity, DHEAS, metanephrine and normetanephrine; thus a non-functional tumor. As there is concern for malignancy for a large heterogeneously enhancing and metabolically active tumor, adrenalectomy was recommended.

He underwent hand-assisted laparoscopic left adrenalectomy that was converted to an open procedure due to the large size of the tumor, large spleen, obese body habitus, and bleeding from an accessory renal vein that was controlled. The tumor did not have invasive features and was removed without violation of the tumor or adrenal gland.

**Fig. 3:**
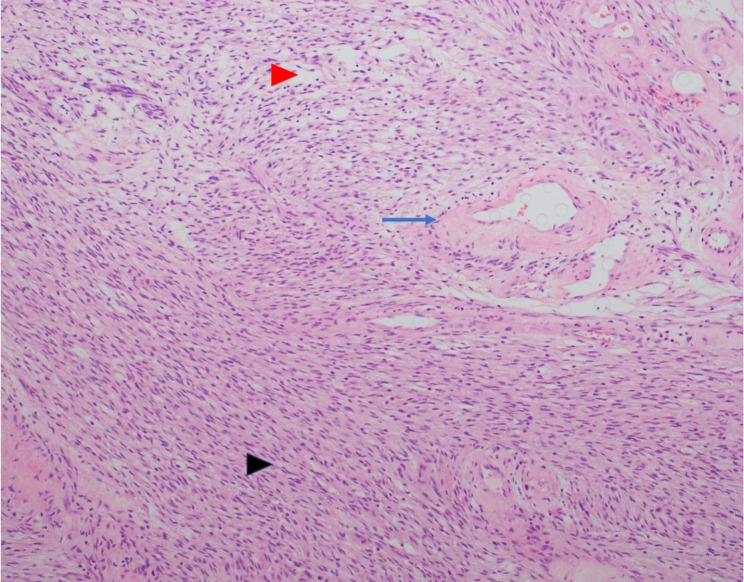
Adrenal schwannoma showing hypercellular Antoni A areas (black arrowhead) and loosely organized Antoni B areas (blue arrowhead), with hyalinized vessels (blue arrow) (hematoxylin-eosin, original magnification X100)

**Fig. 4:**
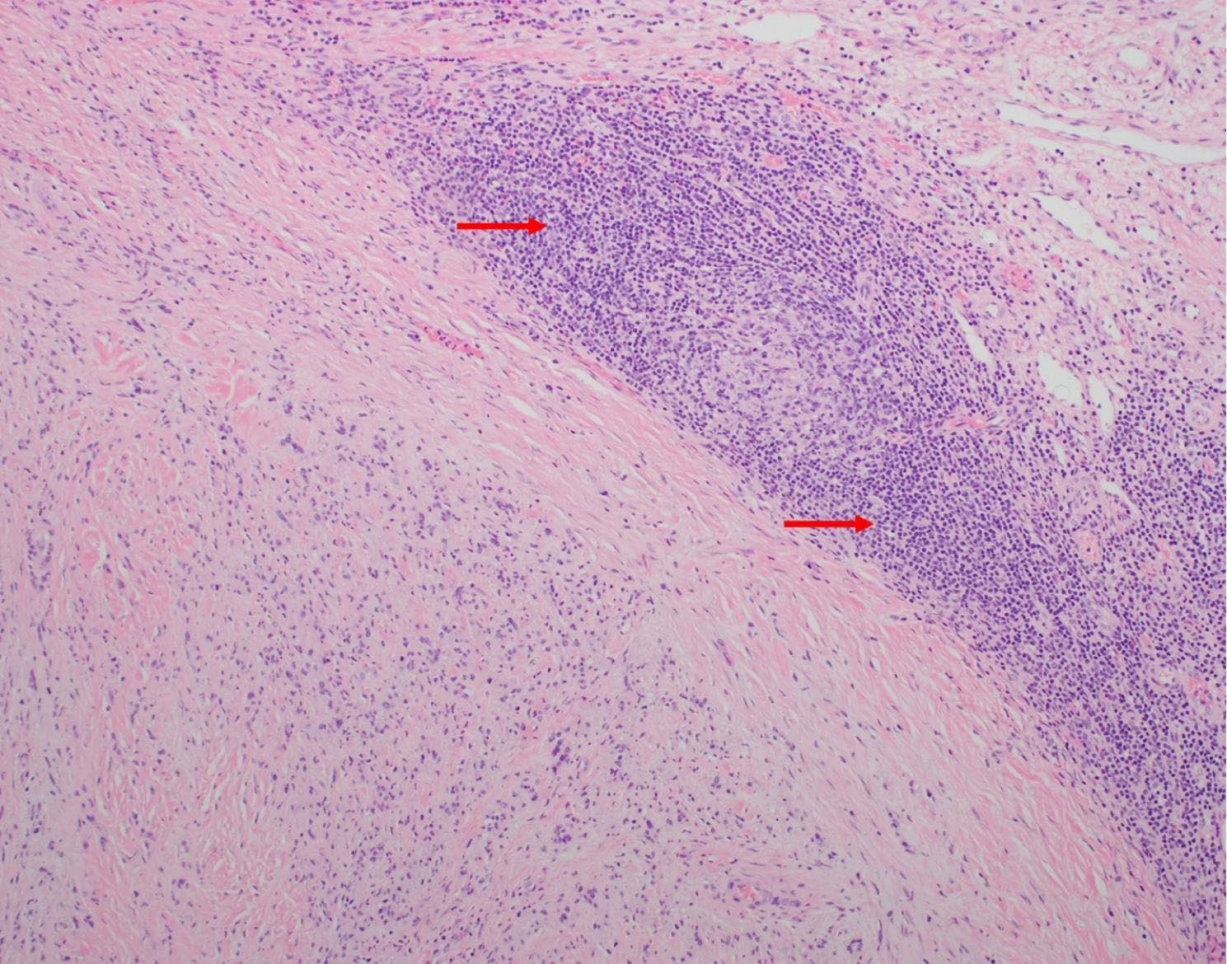
The tumor is composed of monomorphic spindle cells (hematoxylin-eosin, original magnification X100) with the background vasculature showing prominent hyalinization (inset, X200)

**Fig. 5:**
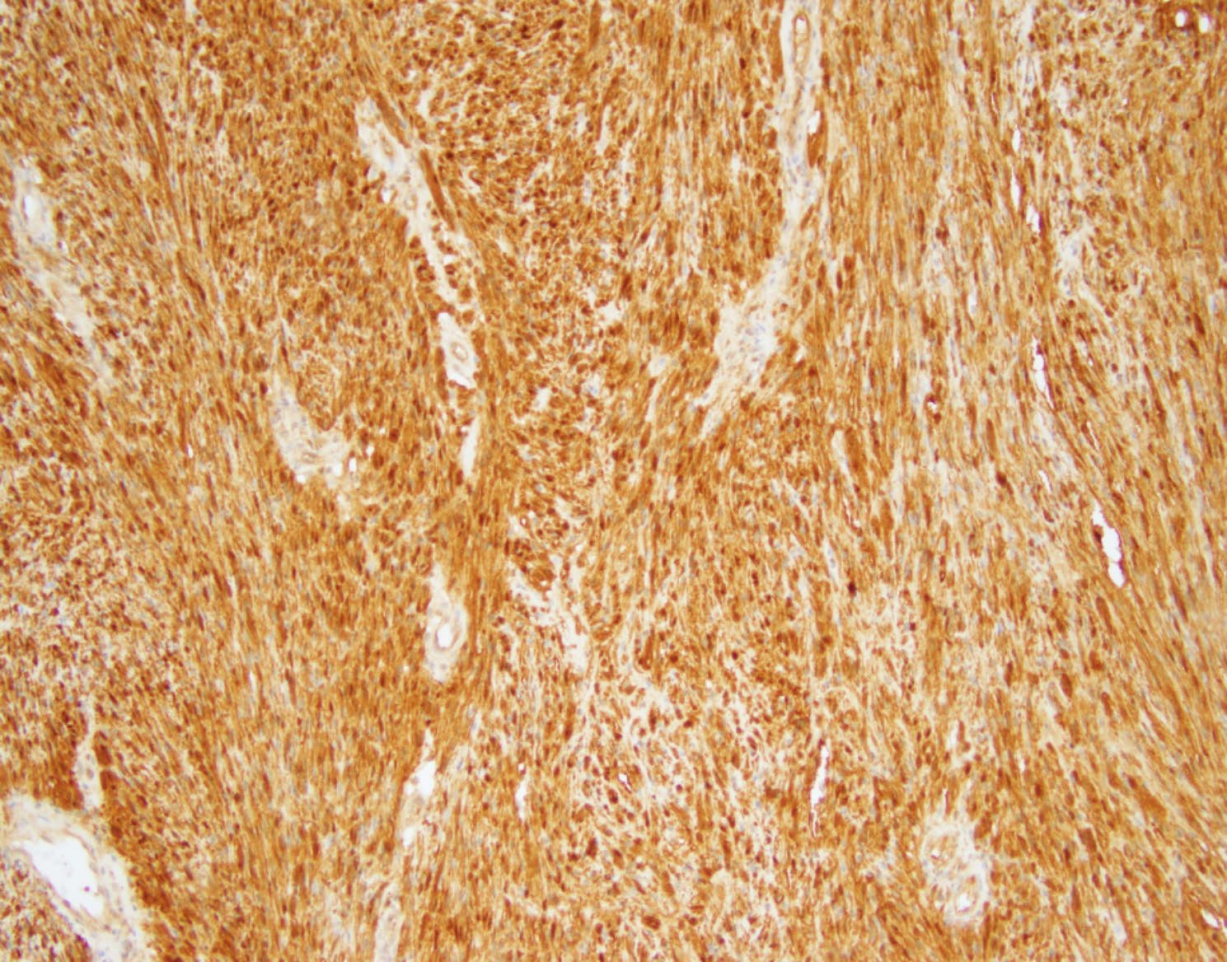
The tumor is well-circumscribed with associated lymphoplasmacytic cuffing (red arrows) (hematoxylin-eosin, original magnification X100)

**Fig. 6:**
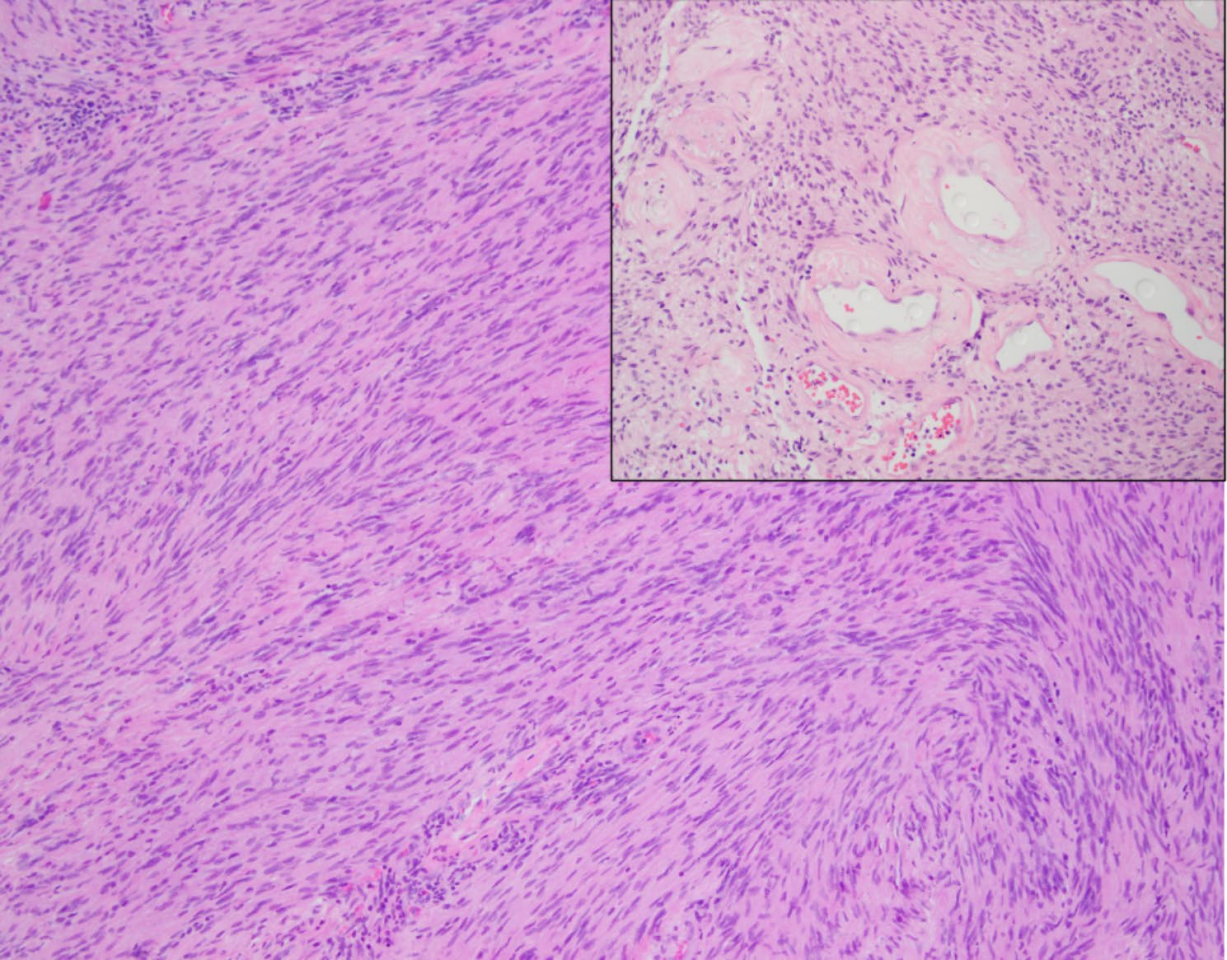
Diffuse nuclear and cytoplasmic staining for S-100 protein (Cell Marque 4C4.9, S-100, original magnification X100)

Microscopic examination showed a well-circumscribed lesion consisting of bland spindle cells with hypercellular (Antoni A) and loosely organized areas (Antoni B) (Fig. 3). Vessels within the tumor were hyalinized (Figs. 3 and 4). Lymphocytic aggregates were seen around the tumor periphery (Fig. 5). The spindle cells were diffusely positive for S-100 (Fig. 6). No atypia, mitotic figures, or necrosis was identified. The final pathology was signed out as adrenal schwannoma (6.1 cm) with an unremarkable adrenal cortex.

## Review of historic institutional pathology

A retrospective analysis of all adrenal pathology ranging from December 2001 to July 2020, including 706 cases of adrenalectomy, revealed two additional cases of adrenal schwannoma. The Institutional Review Board approved this study.

The first case was a 77 year-old female with a left adrenal mass who underwent open left adrenalectomy in 2001 revealing a 13 cm left adrenal ancient schwannoma. Histologic examination showed sheets and fascicles of spindle cells in a background of fibrous tissue. Antoni A and Antoni B areas were seen. Occasional large atypical spindle cells were noted. The spindle cells were positive for S-100, Vimentin, SMA (focally), and negative for EMA, desmin, and HMB-45. No mitotic figures were identified. No clinical or histologic images were available.

**Fig. 7:**
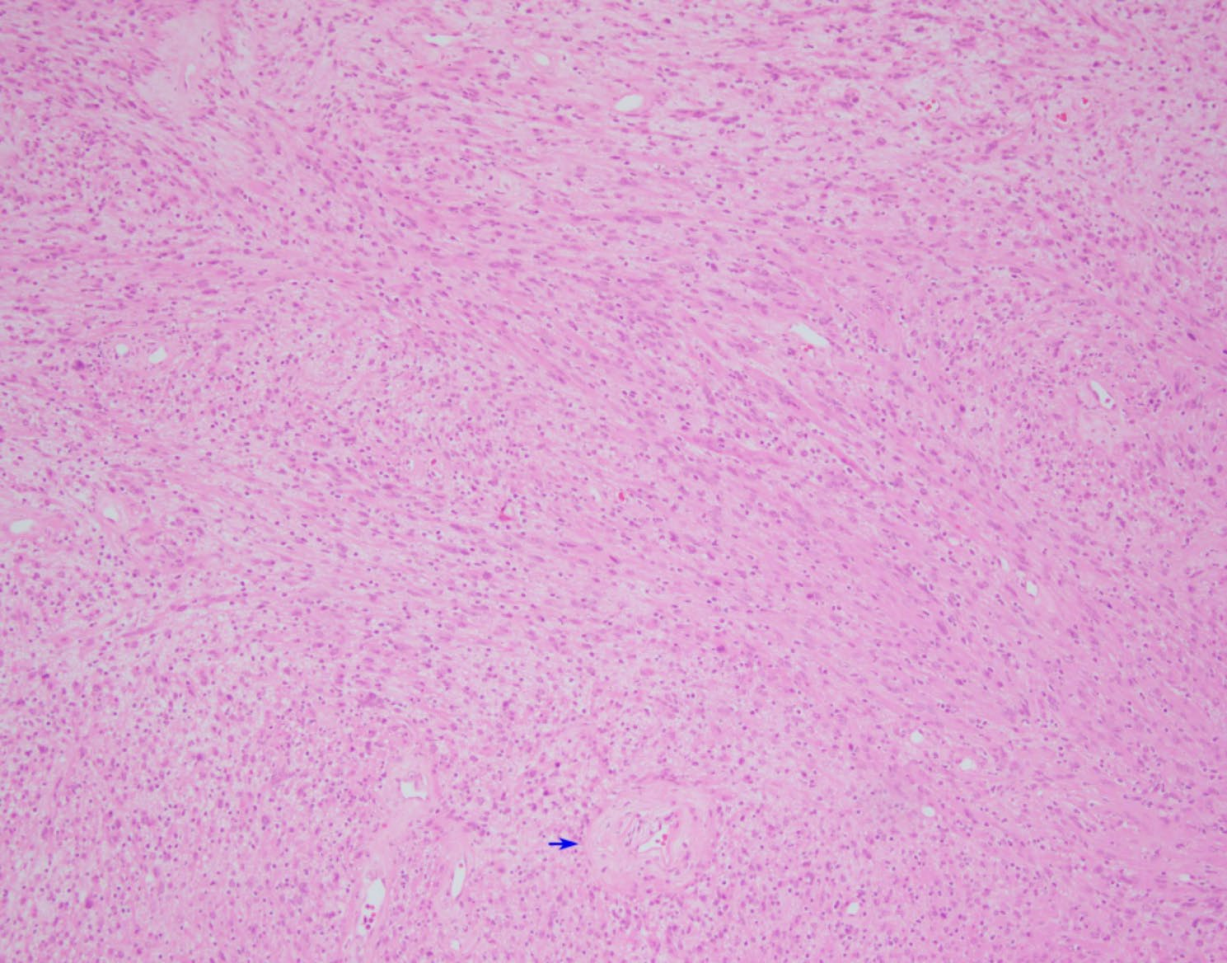
Fascicles of spindle cells in a background of fibrous tissue, occasional large atypical spindle cells are noted demonstrating ancient change. Hyalinized vessels are present (blue arrow) (hematoxylin-eosin, original magnification X100)

**Fig. 8:**
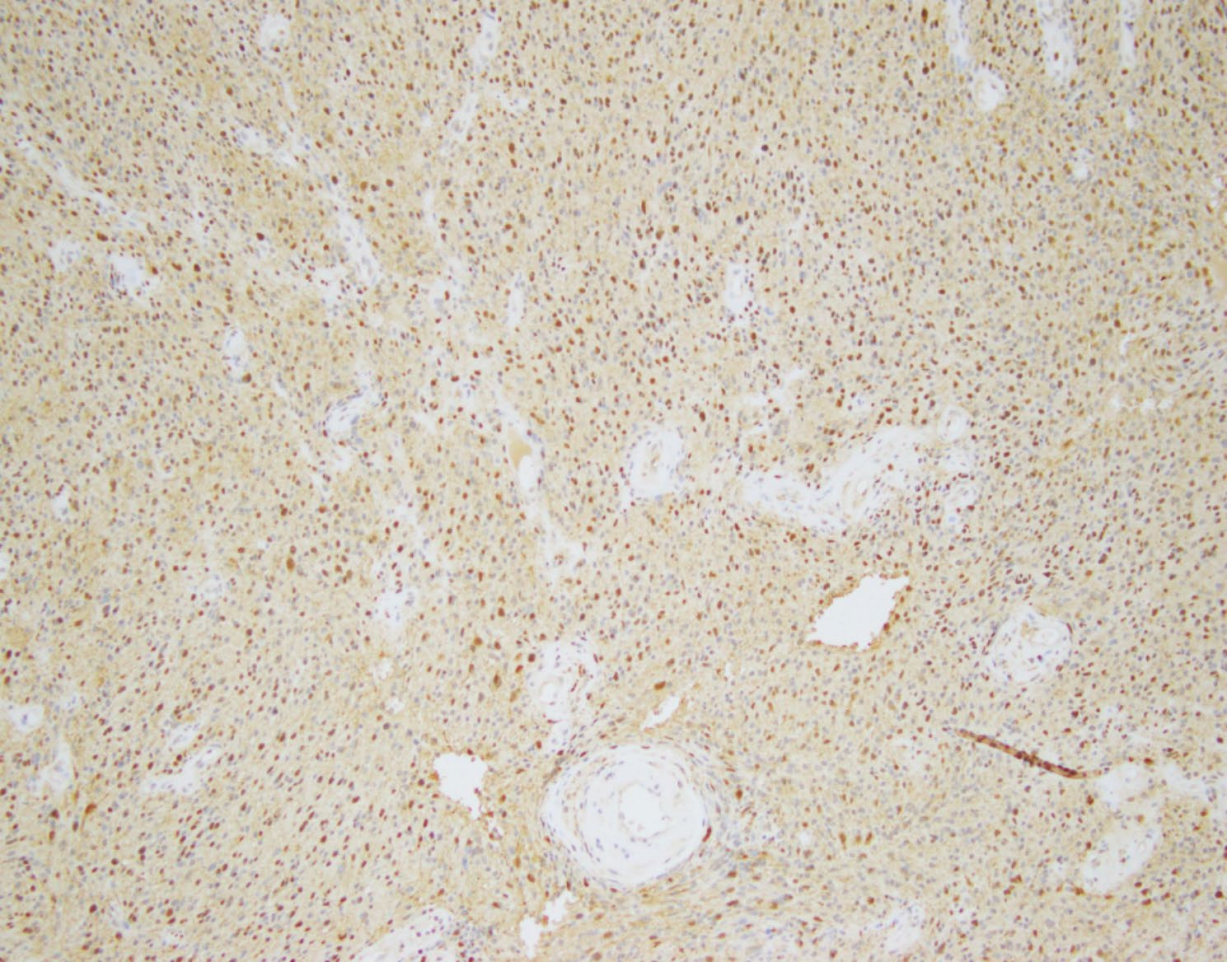
Diffuse nuclear and cytoplasmic staining for S-100 protein (S-100, original magnification X100)

**Fig. 9:**
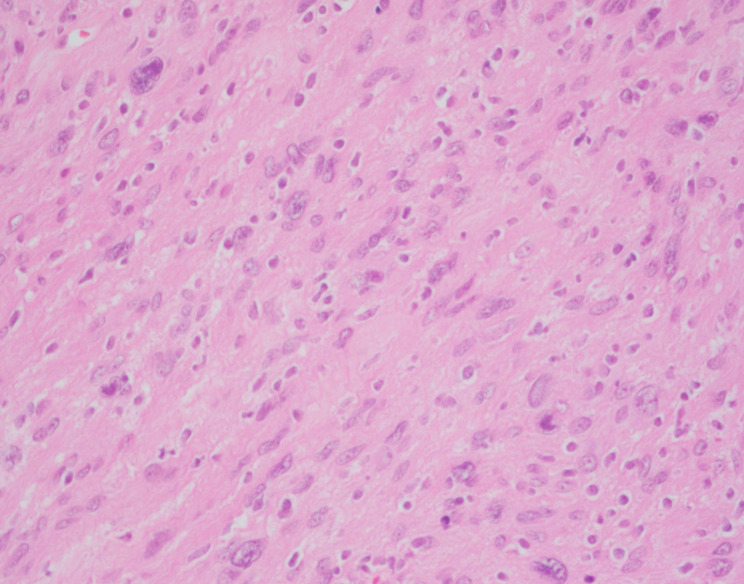
Ancient schwannoma; the tumor cells demonstrate cytologic atypia with nuclear pleomorphism and irregular contours (hematoxylin-eosin, original magnification X400)

The second case was an 81 year-old female with a well-circumscribed left adrenal mass who underwent laparoscopic left adrenalectomy in 2010 revealing a 7 cm left adrenal schwannoma with ancient change. Microscopy revealed a circumscribed lesion composed of fascicles of spindle cells (Fig. 7). Hyalinized vessels were also present. The spindle cells were diffusely positive for S-100, and negative for pankeratin, SMA, HMB-45, and inhibin (Fig. 8). Occasional large atypical cells with nuclear pleomorphism and irregular nuclear shapes were seen (Fig. 9). No clinical images were available.

## Literature review and discussion

Schwannomas are generally considered benign neoplasms of neural crest origin that are difficult to diagnose preoperatively, potentially necessitating surgical excision and or biopsy for proper diagnosis. These tumors most commonly arise from peripheral nerves of the head, neck and extremities 1. Adrenal schwannomas are extremely rare with less than 50 reported cases in the literature 2, 3, 4 and with only 1–3% of schwannomas presenting as retroperitoneal masses 5. Adrenal schwannomas largely present as incidental, non-functional tumors 6. The largest case series reports 31 primary adrenal schwannomas with the majority (87%) of these tumors incidentally discovered with a mean size of 5.7 cm and mean age at diagnosis of 50 years old 2. All are S-100 positive indicating neural crest derivation. There were no recurrences or metastases at an average 54 month follow-up.

There is a paucity of literature regarding FDG PET/CT for adrenal schwannoma with only three single case reports, all showing increased metabolic activity of the adrenal lesion 7, 8, 9. Adrenal schwannoma should be included in the differential diagnosis of a hormonally non-functional mass, even with FDG-avidity. The preoperative differential diagnosis of adrenal lesions includes primary an adrenal cortical neoplasms, pheochromocytoma, myelolipoma, and ganglioneuroma/schwannoma, as well as metastatic disease. In the evaluation of an adrenal lesion, it is of critical importance to rule out pheochromocytoma with biochemical assessment of catecholamine excess with measurement of fractionated plasma metanephrines (metanephrine and normetanephrine), or urine collection for the same. As a catecholamine-producing neuroendocrine tumor of the adrenal medulla, pheochromocytoma must be identified so as to prepare the patient with adequate alpha-adrenergic blockade prior to any surgical intervention, including biopsy, if planned 10. As the leading differential of an FDG-avid mass is malignancy, most patients will proceed to adrenalectomy. A definitive diagnosis of adrenal schwannoma is possible only after histological examination. Immunohistochemically staining for S-100 is helpful for a definitive diagnosis. The histologic differential diagnosis is limited, but a positive S-100 stain and characteristic morphology including alternating cellular areas (Antoni A) and paucicellular areas (Antoni B) allows for a straightforward diagnosis. Keeping schwannoma in mind as a differential diagnosis in the proper clinical setting is important as a biopsy rendering these findings may allow for observation over adrenalectomy, especially for poor surgical candidates.

## Data Availability

Not applicable.
